# Leveraging genetically simple traits to identify small-effect variants for complex phenotypes

**DOI:** 10.1186/s12864-016-3175-3

**Published:** 2016-11-03

**Authors:** K. E. Kemper, M. D. Littlejohn, T. Lopdell, B. J. Hayes, L. E. Bennett, R. P. Williams, X. Q. Xu, P. M. Visscher, M. J. Carrick, M. E. Goddard

**Affiliations:** 1 Faculty of Veterinary and Agricultural Sciences, University of Melbourne, Royal Parade, Parkville, Victoria 3052 Australia; 2 Livestock Improvement Corporation, Cnr Ruakura and Morrinsville Roads, Newstead, Hamilton, 3240 New Zealand; 3 School of Biological Sciences, University of Auckland, 3A Symonds Street, Auckland, 1010 New Zealand; 4 AgriBio, Centre for AgriBioscience, Department of Economic Development, Jobs, Transport and Resources, Bundoora, Victoria Australia; 5 Dairy Futures co-operative Research Centre, AgriBio, 1 Park Drive, Bundoora, Victoria 3086 Australia; 6 La Trobe University, AgriBio, 1 Park Drive, Bundoora, Victoria 3086 Australia; 7 CSIRO Agriculture and Food, Sneydes Road, Werribee, Victoria 3030 Australia; 8 Queensland Brain Institute, University of Queensland, St Lucia, Queensland 4072 Australia; 9 Berghan Carrick Consulting, Moonee Ponds, 3039 Australia

**Keywords:** QTL mapping, Gene expression, Pleiotropy, Complex traits

## Abstract

**Background:**

Polymorphisms underlying complex traits often explain a small part (less than 1 %) of the phenotypic variance (σ^2^_P_). This makes identification of mutations underling complex traits difficult and usually only a subset of large-effect loci are identified. One approach to identify more loci is to increase sample size of experiments but here we propose an alternative. The aim of this paper is to use secondary phenotypes for genetically simple traits during the QTL discovery phase for complex traits. We demonstrate this approach in a dairy cattle data set where the complex traits were milk production phenotypes (fat, milk and protein yield; fat and protein percentage in milk) measured on thousands of individuals while secondary (potentially genetically simpler) traits are detailed milk composition traits (measurements of individual protein abundance, mineral and sugar concentrations; and gene expression).

**Results:**

Quantitative trait loci (QTL) were identified using 11,527 Holstein cattle with milk production records and up to 444 cows with milk composition traits. There were eight regions that contained QTL for both milk production and a composition trait, including four novel regions. One region on BTAU1 affected both milk yield and phosphorous concentration in milk. The QTL interval included the gene SLC37A1, a phosphorous antiporter. The most significant imputed sequence variants in this region explained 0.001 σ^2^_P_ for milk yield, and 0.11 σ^2^_P_ for phosphorus concentration. Since the polymorphisms were non-coding, association mapping for SLC37A1 gene expression was performed using high depth mammary RNAseq data from a separate group of 371 lactating cows. This confirmed a strong eQTL for SLC37A1, with peak association at the same imputed sequence variants that were most significant for phosphorus concentration. Fitting any of these variants as covariables in the association analysis removed the QTL signal for milk production traits. Plausible causative mutations in the casein complex region were also identified using a similar strategy.

**Conclusions:**

Milk production traits in dairy cows are typical complex traits where polymorphisms explain only a small portion of the phenotypic variance. However, here we show that these mutations can have larger effects on secondary traits, such as concentrations of minerals, proteins and sugars in the milk, and expression levels of genes in mammary tissue. These larger effects were used to successfully map variants for milk production traits. Genetically simple traits also provide a direct biological link between possible causal mutations and the effect of these mutations on milk production.

**Electronic supplementary material:**

The online version of this article (doi:10.1186/s12864-016-3175-3) contains supplementary material, which is available to authorized users.

## Background

Genetic variation in complex traits is typically due to thousands of polymorphisms each of which explains a small part (less than 1 %) of the phenotypic variance (σ^2^_P_). This makes it very difficult to identify causal variants [1]. Even with sample sizes > 100,000, genome wide significant associations usually explain < 25 % of phenotypic variance [2]. Bovine milk is an important source of human nutrition and milk production traits (such as milk yield, or fat and protein content) are typical complex traits where many loci and environmental effects influence phenotypes. Although some mutations with relatively large effects on milk production traits have been identified (e.g. DGAT1 [3]), the majority of the genetic determinants that cause variation in milk production traits remain unknown. This is because the remaining genetic determinants explain only a small percentage of phenotypic variance for these traits and studies typically lack statistical power to confidently identify these loci. The challenge is to identify the causative mutations that underpin these QTL of small effect on a genome wide scale.

With the aim of achieving this, we describe a new approach using secondary, potentially genetically simpler, traits, where effects of mutations might be expected to be larger than for the complex trait, to map causal variants for milk production traits. Although other studies have used related phenotypes or gene expression to verify QTL for complex traits, few studies use these data during the QTL discovery for small-effect (<1 % σ^2^_P_) loci or when the phenotypic correlation between the traits is low [4]. We used a dataset of 11,527 genotyped cows with phenotypes including milk production, and also secondary phenotypes for a subset of 400 of these cows including 16 detailed milk composition phenotypes (individual proteins, mineral concentrations), and gene expression on a separate sample of 371 cows. The aim was to use these secondary phenotypes to assist in identification and precise mapping of loci with small effects (<1 % σ^2^_P_) on milk production. The power of the method is demonstrated by the identification of a QTL that affected both milk yield and phosphorous concentration in milk, centred on the gene SLC37A1, a phosphorous antiporter. The most significant imputed sequence variants in this region explained 0.001 σ^2^_P_ for milk yield, and 0.11 σ^2^_P_ for phosphorus concentration.

## Results and discussion

For both milk production traits and secondary traits (composition traits including proteins and minerals, Table 1 and Additional file 1: Table S1), we estimated haplotype effects for sliding windows of 250 kb across the genome. Haplotypes were derived from SNP genotypes (632,003 genome wide SNP in 11,527 cows) and the effects of these haplotypes on the traits were estimated with BayesR [5, 6]. We identified regions that show high variance in estimated haplotype effects for both milk production traits and secondary phenotypes. There were 8 regions that contained a QTL for a milk production and composition trait (chi-squared test *P* < 0.05 Bonferroni-corrected, Table 1). As a negative control we analysed a trait with no direct relationship to milk composition (stature, see methods) and found no significant overlap between regions with QTL for stature and milk composition traits.

**Table 1:** Genomic regions with overlapping QTL between milk production and composition traits

BTAU6	Region (Mb)	Milk production traits	Milk composition traits
1	144.2–144.65	MY, F%, P%	phosphorus
3	7.7–8.15	P%	IgG
6	37.4–37.95	F%, P%	lactose%
6	87.15–87.65	MY, PY, P%	κ-casein
11	103.1–103.55	FY, MY, PY, F%	β-lactoglobulin
14	1.60–2.25	FY, MY, PY, F%, P%	Ca, S, P, κCN
17	56.35–56.6	FY, PY	calcium
20	33.35–33.75	P%	lacto-peroxidase

Milk production traits are FY = fat yield (kg/lactation), MY = milk yield (L/lactation), PY = protein yield (kg/lactation), F% = fat percentage in milk, P% = protein percentage in milk. Milk composition traits include phosphorus (P, mg/kg), IgG (mg/g), κ-casein (κCN, mg/g), β-lactoglobulin (mg/g), calcium (Ca, mg/kg), sulphur (S, mg/kg) and lacto-peroxidase (mg/g) concentration in milk

The 8 regions include several already identified as important for milk production, including *ABCG2* (BTAU6, 38 Mb) [7], the casein complex (BTAU6, 87 Mb), *PAEP* (formally known as β-lactoglobulin, *LGB*; BTAU11), and *DGAT1* (BTAU14) [3]. These 4 regions can be viewed as positive controls, and in at least 3 of the 4, the component trait would help identify the correct gene. For instance, on chromosome 11, mutations near β-lactoglobulin affect the expression of the gene and hence the concentration of the β-lactoglobulin protein in milk [8]. For the 4 novel regions, there are promising candidate genes with direct links to the composition traits, including several IgG receptors (e.g. *FCGR2*) on chromosome 3 and a calcium transporter (*ATP2A2*) on chromosome 17.

**Fig. 1:**
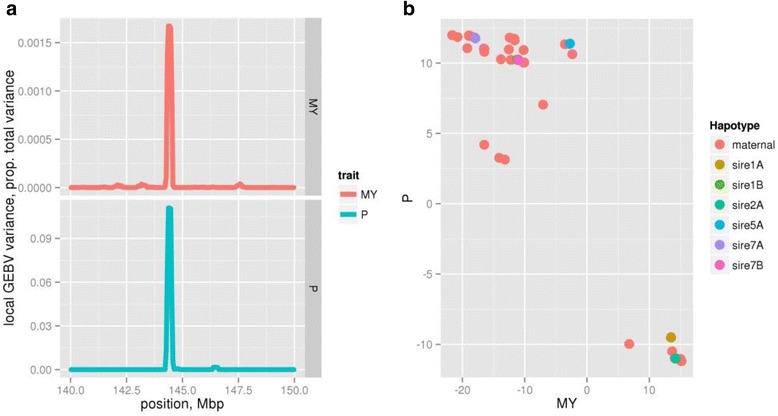
**a** The overlapping QTL region in milk yield (MY), predicted from 11,527 animals, and milk phosphorus concentration (P), predicted from 444 animals, on chromosome 1 at approx. 144.4 Mbp. **b** The estimated haplotype effects for genetic merit of phosphorus concentration (mg/kg) and milk yield (L/lactation) for haplotypes spanning 144.25–144.5 Mbp on chromosome 1. Cows measured for milk composition traits had a strong family structure and were from one of 8 sire families. Figure 1b shows the (non-identical) maternal haplotypes in pink, while paternal haplotypes were randomly assigned to either haplotype A or B from each sire. Note that although all animals were from 8 half-sib families, sires that carried identical haplotypes effects were assigned to the first sire where this haplotype was observed

We investigated two regions in detail. The first is a novel region on chromosome 1, where Fig. 1a shows the alignment of the variance in estimated genetic merit for milk yield and phosphorus concentration centred on (approx.) 144.4 Mbp. Figure 1b shows that the haplotype effects for the 444 cows measured for the milk composition traits clearly separate into two groups, supporting the hypothesis that these haplotypes represent two alleles affecting both traits. To identify possible causal variants, we imputed genome sequence from the 1000 bull genomes project [9] into the region and used EMMAX [10] to conduct association studies. Due to the small-effect of the locus on milk production traits, the analysis of production data used a multi-trait meta-analysis [11] strategy while a standard association test was conducted for milk phosphorus concentration.

**Fig. 2:**
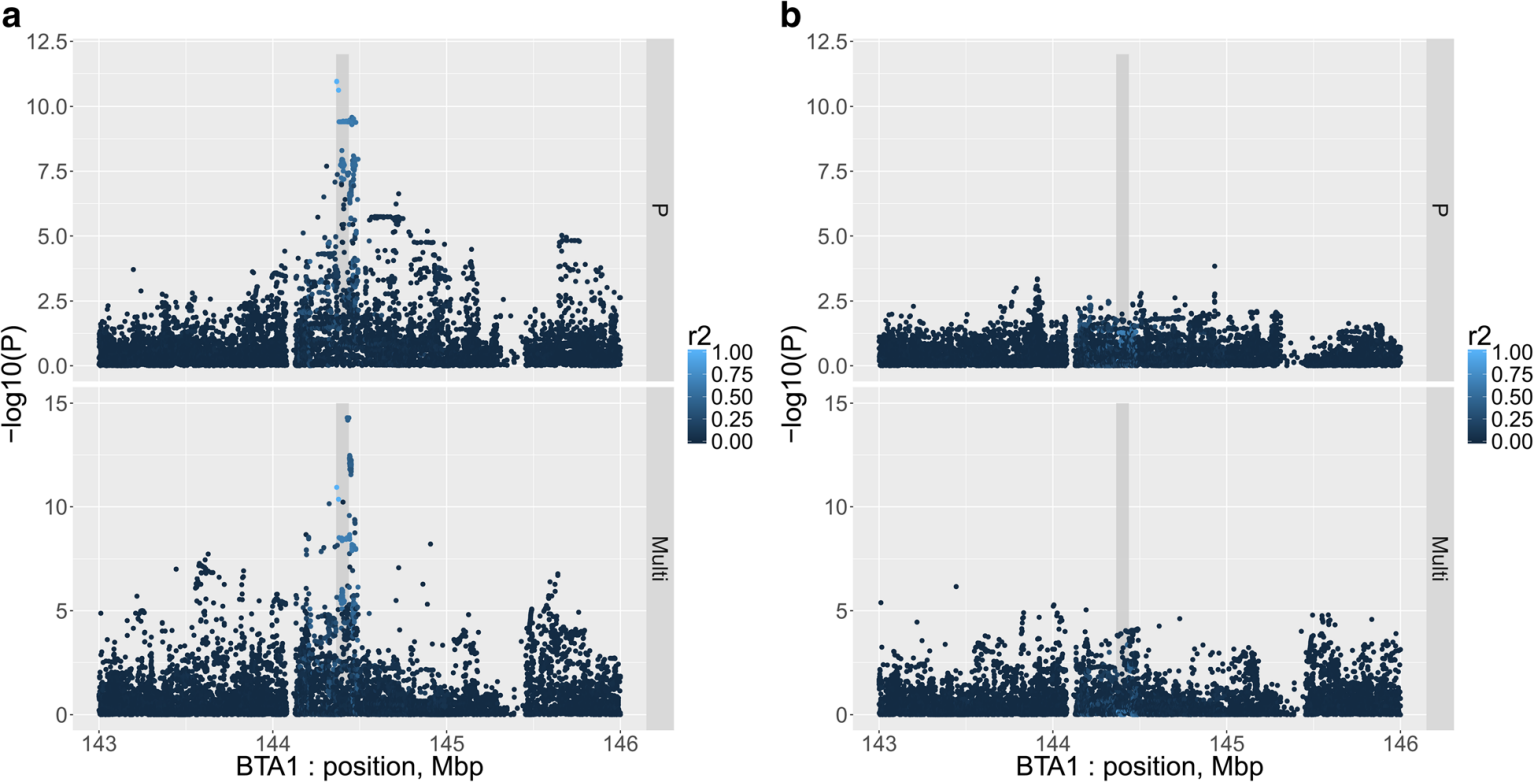
Phosphorus (top) and the multi-trait (bottom) association analysis between sequence variants and milk production traits near *SLC37A1* (grey region), without fitting covariables (**a**) and fitting rs109254133 as a covariable (**b**). The legend indicates the LD (r^2^) between the fitted variant and all other variants

Peak significance for phosphorus concentration was observed for two variants mapping to intron 2 of the *SLC37A1* gene (rs109254133 and rs208161466; *P* < 1 × 10^−10^), both of which were also highly significant in the multi-trait meta-analysis (*P* < 1 × 10^−10^) and in complete LD in the sequenced animals (Additional file 1: Table S2). When either of these variants was fitted as a co-variable in subsequent association analyses, there were no remaining highly significant (*P* < 5 × 10^−8^) sequence variants in the region for either phosphorus concentration or in the multi-trait analysis (Fig. 2). The rs109254133 variant explained 0.001 σ^2^_P_ for milk yield, and 0.11 σ^2^_P_ for phosphorus concentration. The effect on milk yield was confirmed using a sample of a different breed of cows (Jersey) (*P* = 0.003), where rs109254133 explained 0.002 σ^2^_P_ in milk yield.

Since rs109254133, rs208161466 and all other less significantly associated polymorphisms (*P* < 1 × 10^−9^ for phosphorus concentration) were non-coding, we performed association mapping for *SLC37A1* gene expression with PLINK [12] (http://pngu.mgh.harvard.edu/purcell/plink/). Using high depth mammary RNAseq data from a separate group of 371 lactating cows, we confirmed a strong eQTL for *SLC37A1*, with peak association demonstrated for the same two SNP highlighted from analyses of the milk traits (rs109254133 & rs208161466, *P* = 3.6 × 10^−18^; Additional file 1: Figure S1 and Table S2). These data strongly support *SLC37A1* as the causative gene for the observed variation in these phenotypes. *SLC37A1* functions as a phosphorus:glucose-6-phosphate antiporter [13]. That is, it transports glucose-6-phosphate in one direction and phosphorus in the other. Glucose is needed for lactose synthesis in mammary cells and lactose controls milk volume because it is the major osmotic component of milk [14]. In support of an antiporter hypothesis the allele that increases *SLC37A1* expression (the derived ‘T’ allele [15] for rs109254133) increases milk yield (+37.6 L/lactation) and decreases phosphorus concentration (−41.8 mg/kg). Although neither rs109254133 nor rs208161466 appear evolutionarily conserved, their uniform association across phenotypes (and independent datasets) highlights these variants for future functional investigation. This region shows a clear link between gene function, two related phenotypes with moderate effect QTL (milk phosphorus concentration and gene expression) and a complex trait with a QTL for explaining as little as 0.001 σ^2^_P_.

The second region investigated was located near the casein complex on chromosome 6, where there are four casein-encoding genes (α_S1_-, α_S2_-, β- and κ-casein) in a 300 kb region. The casein proteins constitute about 80 % of the protein content in bovine milk. Although protein polymorphisms have been described in these gene products for many years, their association with milk production traits including milk protein yield remains uncertain [10]. We imputed genome sequence into the region to conduct association and eQTL studies as for the analysis of chromosome 1. The highest association in component traits was for κ-casein concentration, where 134 variants were in strong LD (i.e. within one –log_10_ unit of the top variant, *P* < 7.7 × 10^−11^). The κ-casein eQTL analysis revealed a strong association for 6 variants (*P* < 3.3 × 10^−21^; Additional file 1: Figure S2), three of which were also genotyped in the 1000 bull genomes dataset [11] and were highly significant for κ-casein concentration. The variant most highly associated with the expression of κ-casein (rs209251505) also increased concentration of the protein in milk. This variant is located 13.859 Kb downstream of the gene encoding κ-casein (*CSN3*).

**Fig. 3:**
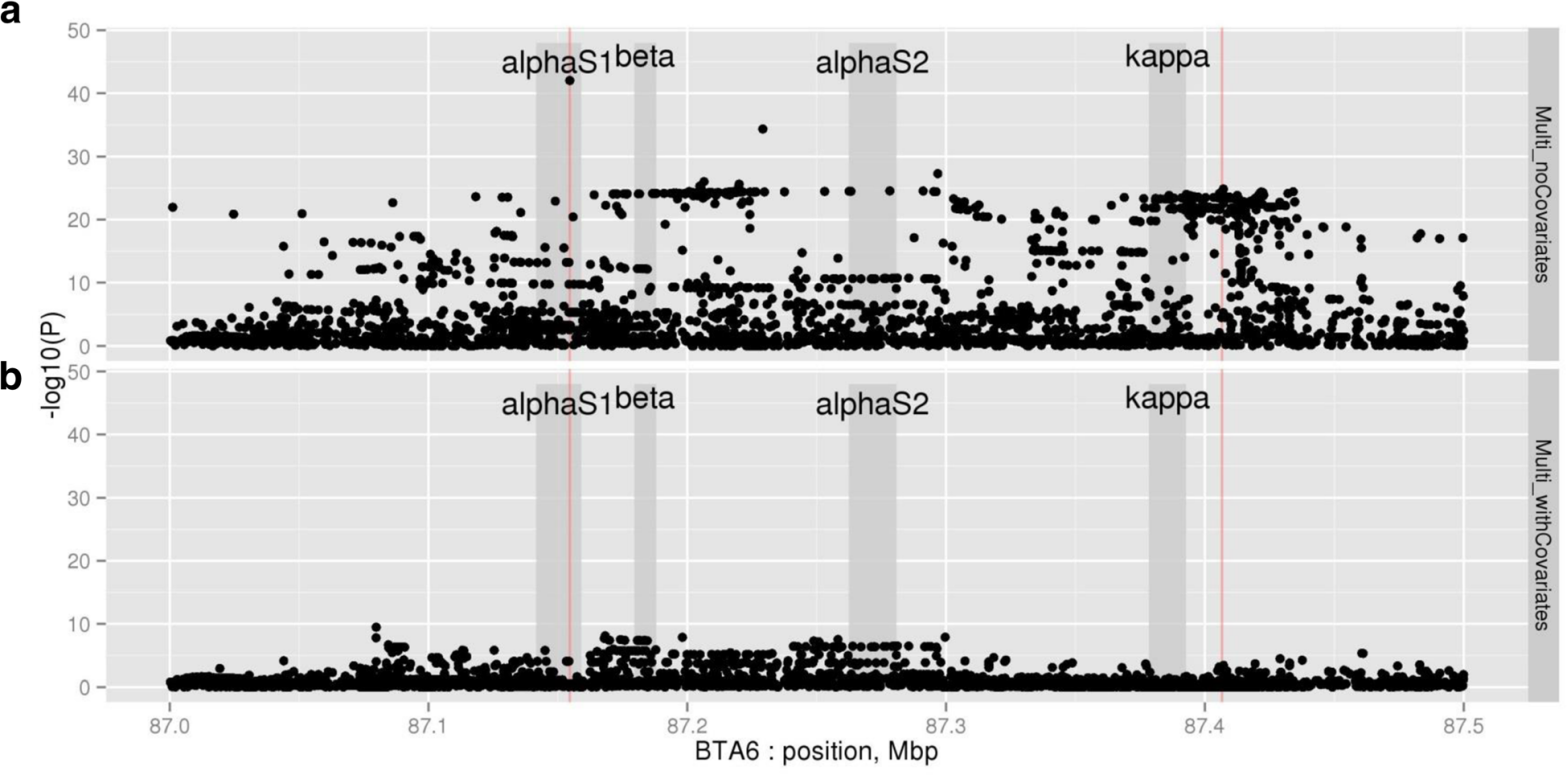
Multi-trait association analysis between sequence variants and milk production traits near the casein complex, without fitting covariates (**a**) and fitting rs109193501 and rs209251505 (**b**). Positions for the fitted variants are indicated by the vertical lines and the open reading frames of the casein genes are shaded

The rs209251505 variant did not remove the entire QTL signal in the casein complex region. To determine if we could use the protein concentration phenotypes to distinguish between candidate genes in close proximity, we fitted rs209251505 as a co-variable in all analyses of milk production and composition traits. The most significant trait was α_S1_-casein concentration, where 18 variants were significant (*P* < 2.0 × 10^−5^; Additional file 1: Figure S3). Neither these nor any other variants significantly affected the expression of the gene encoding α_S1_-casein (CSN1S1; *P* > 1 × 10^−3^, Additional file 1: Figure S2). However we identified a SNP from the list of 18 candidates that was the most significant variant in the Holstein multi-trait analysis of milk production traits (rs109193501; *P* = 1.0 × 10^−26^; Additional file 1: Table S3). This SNP is located within an intron of CSN1S1 and its effects validated for P% in the Jersey cow population (*P* = 1.1 × 10^−18^, after fitting rs209251505 as a co-variable). Thus, the QTL appears to affect α_S1_-casein production but its precise mechanism is unclear as the variant is not associated with a change in gene expression. When both the κ- and α_S1_-casein variants were fitted as co-variables (rs209251505 & rs109193501), only weak associations for the multi-trait analyses remained (*P* > 1 × 10^−10^; Fig. 3).

Thus the casein region appears to have at least two independent QTL, represented by rs209251505 and rs109193501, which contribute to variation in milk production traits. The first, rs209251505, was estimated to explain 0.003 σ^2^_P_ for P% and 0.08 σ^2^_P_ for κ-casein concentration (after fitting rs109193501; Additional file 1: Table S4). The results suggest that one allele of this polymorphism, or one of those in high LD with it, increases expression of *CSN3* causing increased synthesis of the κ-casein protein, and thus an increase in κ-casein and total protein concentration in milk. The second variant, rs109193501, was estimated to explain 0.01 σ^2^_P_ for P% and 0.04 σ^2^_P_ for α_S1_-casein concentration (after fitting rs209251505; Additional file 1: Table S3), although the precise mechanism by which it may modulate abundance is unclear. Our study indicates little effect of the previously reported coding polymorphisms [9], suggesting that the previous inconsistencies in reports were due to variation in LD between studies.

## Conclusions

These analyses demonstrate the use of information from genetically simple traits (secondary traits) to identify QTL explaining as little as 0.001 σ^2^_P_ in milk production traits. By leveraging the larger effect of the loci in the genetically simple traits, we were able to use records on approximately 400 individuals to confidently identify these loci. We attempted to identify the causative mutations underlying these QTL using imputed sequence data but there were many potential candidates in high LD and no known functional roles in the genome. We conclude that using secondary, and genetically simple, traits is a viable alternative to increasing sample size for the identification of small-effect QTL, particularly where it may take several years to accumulate sufficient additional data to attain the required increases in statistical power. Our results also show that phenotypes with direct biological links to gene function are useful to distinguish between candidate genes in close proximity.

## Methods

### Overview of data and analyses

This paper uses eight datasets to (1) conduct QTL mapping with BovineHD (high density) SNP in milk production and component traits, (2) conduct association studies with imputed sequence variants in target regions for milk production and component traits, and (3) conduct an eQTL analysis with sequence variants in target regions to identify likely causal variants. Many of the datasets represent exact data or expanded datasets from previously described analyses and Additional file 1: Table S5 shows the number of animal records used in this analysis for each data type and their references (where relevant). New data includes the 16 milk component traits and its collection was approved by the Department of Primary Industries Ethics Committee. This is the first analysis to consider the two completely independent data sources of sequence variants from a global initiative (i.e. the 1000 bull genomes dataset) and from a dataset generated in New Zealand by the Livestock Improvement Corporation. Further details on data and the analysis are given below.

### Data collection for milk component traits

There were 728 cows whose combined morning and afternoon milk samples were measured for lactose, mineral (calcium, potassium, magnesium, sodium, phosphorus, sulphur, zinc) and protein (lactoperoxidase, lactoferrin, immunoglobulinG, alpha-lactalbum, beta-lactoglobulin, kappa-casein, alpha-S1-casein, beta-casein) concentrations. Traits were measured 1 or 2 times with a 6 week interval between samplings. Details for the number of records, trait means and measurement units are given in Additional file 1: Table S1. Minerals were assayed by microwave acid digestion of homogenised milk samples in a mixture of nitric acid and hydrogen peroxide and measuring the digestant using Inductively Coupled Plasma Emission Spectroscopy (all minerals except zinc) and atomic absorption spectrophotometry (zinc only). Major milk proteins (alpha-lactalbum, beta-lactoglobulin, the 3 casein types) were measured using capillary zone electrophoresis [16] with minor proteins (lactoperoxidase, lactoferrin, immunoglobulinG) quantified by HPLC.

### Phenotype and genotype preparation for milk component traits

The model fitted to the data aimed to correct phenotypes for non-genetic effects. ASReml [17] was used to fit the following model to each trait: trait = mean + breed_i_ + age^4^ + dim^4^ + HYS_j_ + PE_j_ + anim_j_ + e_j,k_; where i = breed code (8 levels, accounting for degrees of Holstein, Jersey and unknown ancestry); age^4^ and dim^4^ = covariates of cow age (age) and days-in-milk (dim) fitted as 4th order polynomials; PE_j_, anim_j_ and HYS_j_ = random effects for permanent environment [PE ~ N(0,σ^2^_PE_)], additive genetic [anim ~ N(0,σ^2^_A_)] and herd-year-season (HYS_j_, HYS ~ N(0,σ^2^_HYS_)] for cow j and e_j,k_ is the residual for measurement k from cow j. Thus a phenotype for animal j was ∑_n_(PE_j_ + anim_j_ + e_j,k_)/n, where n is the number of records for cow j. Only cows with 2 records were used in the final analysis (i.e. up to 444 animals). Animals had real and imputed Illumina BovineHD BeadChip genotypes for 632,003 SNP. Quality control procedures and imputation were carried out as part of the larger population of genotyped bulls and cows (see below) following [5]. Quality checks included pruning of SNP on the basis of their GenTrain score (Gen-Call > 0.6) and removal of SNP with less than 10 copies of the rare allele in the larger population. Imputation used Beagle v3 [18]. The cows included in this dataset had a strong family structure and most were from one of 8 sire families (Additional file 1: Figure S4).

### Data for milk production traits

The milk production data is the Holstein reference of 8,478 cows and 3,049 bulls as described by Kemper et al. [6]. Briefly, these are animals evaluated under Australian conditions for 5 milk production traits; milk yield (L/lactation), fat yield (kg/lactation), protein yield (kg/lactation), fat percentage in milk (%) and protein percentage in milk (%). Traits were obtained from the Australian Dairy Herd Improvement Scheme as either trait-deviations (for cows) or daughter-yield deviations (for bulls) which are phenotypes pre-corrected for non-genetic effects. Some of these records are highly accurate as they are the culmination of up to 6 lactations or, in the case of bulls, many 1000’s of daughter records, potentially with multiple lactations contributing to each daughter record. The Jersey cow population used for validation of the variants in the latter stages of the association study is the reference dataset of 3,917 cows from Kemper et al. [6] where the phenotypes are trait-deviations for the traits as described above for the Holstein animals. All animals had real and imputed Illumina BovineHD BeadChip genotypes for 632,003 SNP which had passed quality control procedures [5].

### Identification of QTL regions with HD SNP genotypes

QTL were identified in milk production and component traits using regions showing high variance in local genomic estimated breeding values (GEBV, i.e. genetic merit) [19]. Variance in local GEBV were obtained for milk production traits from Kemper et al. [6] using the Holstein-only reference population of 11,527 bulls and cows analysed with the weighted BayesR procedure. This analysis weighted bull and cow records to account for heterogeneous error variance of the data and was found to have moderate-to-high predictive value for overall genetic merit (accuracy = 0.58–0.88) [6]. Thus high variance in local GEBV aimed to identify genomic regions underlying variation in the predicted genetic merit. From Kemper et al. [6], variance in local GEBV are calculated as the variance in **Wv**, were **W** is a matrix of SNP genotypes for the reference population in a 250 kb region and **v** is the SNP effect estimated by BayesR. The local variance in GEBV has the advantage of accounting for the haplotype structure of the data and analysis of small regions (sliding windows of 250 kb) overcomes, in part, problems associated with simultaneous fitting of all variants (e.g. splitting of QTL effects between adjacent SNP in strong LD [20]). Windows of 250 kb were chosen to represent haplotypes segregating in the population prior to breed formation [21]. QTL were defined in milk production traits as the 2 % of the genome with the highest variance in local GEBV. The highest 2 % of windows represent about 90 % of the total cumulative window variance in each trait.

QTL mapping for component traits was also conducted using BayesR [5, 6] and variance in local GEBV. As the heritability of these traits was unknown (and could not be estimated accurately due to the strong half-sib structure in the data), we assumed SNP effects came from a mixture of normal distribution with variance equal to 0, 0.00005, 0.0005 and 0.005 of the phenotypic variance (σ^2^_P_). Local GEBV were calculated as described above from the estimated SNP effects [6]. Milk composition traits showed a range of genetic architectures, with the largest QTL (defined as the 0.1 % of windows explaining the highest variance) explaining > 95 % of the cumulative variance for some simple traits (Grp I traits; Additional file 1: Figure S5) but < 25 % of the total in more complex traits (Grp II traits; Additional file 1: Figure S5). Only the largest QTL for each trait were explored further, where these QTL were investigated for co-location with QTL from milk production traits. Although more formal approaches for declaring QTL under Bayesian frameworks are available, e.g. the calculation of Bayes factors [22], the approach taken here could be applied directly to available data and formal testing used a chi-squared test for independence (see below).

We tested the hypothesis that QTL for milk production traits are independent of QTL for milk composition traits. Thus the expectation was that there should be no overlap between these two sets of QTL. That is, if we select 2 % of the genome with milk production QTL, 0.1 % of the genome with QTL for component traits and there are 10,015 independent windows, then we expect < 1 window overlapping between the two sets (0.02 × 0.001 × 10,015 windows < 1 window). Since the QTL analysis used sliding windows of 250 kb with 50 kb between adjacent windows, we performed the test on the average number of overlapping QTL from each set of non-overlapping windows. The chi-squared test with Bonferroni corrected P-value [0.05/(16 component traits x 5 milk production traits)] tested if the number of significant overlapping QTL regions was more than expected by chance (*P* < 0.05). As a negative control, we also tested the overlap between milk component QTL and a trait with good prediction accuracy but no known relationship to milk component traits. The trait selected was stature (accuracy = 0.54) [6] and, as expected, there was no significant overlap between stature QTL and the largest QTL identified for the 16 milk component traits. Chi-squared tests for all trait pairs with co-locating QTL are given in Additional file 2.

### Imputation of sequence variants and association study in targeted regions

The two regions were chosen for association studies with imputed to full sequence variants. These regions were the most promising novel finding (BTA1:144.4Mbp) and an example of a region near the casein complex (BTA6:87.5Mbp) which has several genes encoding for the major milk proteins. Imputation used phased Holstein variant calls (*n* = 260) from run4 of the 1000 bull genomes project [9] and Minimac2 [23], where SNP used for imputation were quality checked for concordance with 800 K genotypes [24]. Imputed regions included a minimum of 4 Mb surrounding each QTL and focus on either 28,474 (Chr1:143-146Mbp) or 4,527 (Chr6:87–87.5Mbp) variants in the target regions. Sequence variants from the 1000 bull genomes includes bi-allelic SNP and and small bi-allelic indels. Variants with minor allele frequency > 0.001 were tested for association with the milk production and composition traits (from Table 1) using genotype probabilities in EMMAX [11] and an identity-by-state matrix constructed with 800 K genotypes. Association tests for milk production traits in Holstein bull and cow datasets were conducted separately (to minimise the effect of the heterogeneous error variance of these two data types), and then combined assuming a *n* degree-of-freedom for a chi-squared test statistic where the test statistic for each variant was given by ∑_n_t^2^ (where *n* is the number of t-statistics included in the test [11]). The multi-trait analysis only used the milk production traits identified as containing QTL (i.e. those identified Table 1, for each region). Analyses fitting SNP covariates used the same procedure as above and the covariate option in EMMAX. Validation of sequence variants using Jersey cattle used Jersey cow genotypes and phenotypes (*n* = 3917) as described by Kemper et al. [6] and consisted of SNP genotypes for 632,003 SNP. Sequence imputation used Minimac2 [22], as above, and phased Jersey animals (*n* = 61) from the 1000 bull genomes [9] as the imputation reference.

### eQTL data collection and analysis

Expression QTL analysis was conducted using imputed genomic sequence in conjunction with a mammary RNA sequence dataset representing 406 lactating cows. These data comprised an expanded dataset to that described previously [25]. Briefly, samples were derived by mammary tissue biopsy and total RNA libraries prepared for 100 bp paired end sequencing on the Illumina HiSeq 2000 instrument. Library preparation and sequencing was performed by NZ Genomics Limited (NZGL; Auckland, New Zealand) or the Australian Genome Research Facility (AGRF; Melbourne, Australia). Sequence reads were mapped to the UMD3.1 genome using Tophat2 (version 2.0.12) [26], yielding an average of 88.9 million mapped read-pairs per sample. Expression phenotypes representing *SLC37A1*, *CSN1S1*, and *CSN3* were quantified using v1.14.0 of DESeq [27], representing variance stabilised read counts corresponding to gene structures from Ensembl gene set release 77.

RNAseq animals were genotyped using the Illumina BovineHD BeadChip (*N* = 377), or Illumina SNP50k BeadChip (*N* = 29), with the latter cohort imputed to the BovineHD BeadChip prior to sequence imputation using v4 of Beagle [18]. These data were then merged with an additional variant set called directly from the RNAseq alignments, representing a high confidence, quality-filtered consensus set called using GATK HaplotypeCaller (v3.1) and Samtools (v1.2) [28, 29]. Whole-genome sequence imputation was performed using a sequence reference population of 556 animals described elsewhere [25]. Briefly, genome sequence variants were identified using GATK HaplotypeCaller (v3.1) and phased using Beagle (v4) [18, 28]. Variants with initial allelic R^2^ values > 0.95 in the reference population were retained and imputed into the target population using Beagle (v4) [18]. Any variants in the target population with imputation R^2^ values < 0.70, and minor allele frequency < 0.001 and Hardy-Weinberg thresholds of *P* < 1 × 10^−10^ were removed from further analysis. Plink (v1.90) [12] was used to test the association between sequence variants in the QTL regions and the normalised expression phenotypes described above. BovineHD BeadChip genotypes in conjunction with the identity by state and multidimensional scaling procedure implemented in Plink (v1.90) [12] to calculate population structure covariates for inclusion in the SNP association models. Ten covariates were fitted in these models, representing a practicable number of covariates which together explained > 50 % of the genotypic variation. Models also included a single fixed effect to account for differences in cohorts/sequencing facilities. The sequence intervals comprised 22,263 variants for analysis of *SLC37A1* (Chr1:143-146Mbp), and 3,169 variants for analysis of *CSN3* and *CSN1S1* (Chr6:87–87.5Mbp). The eQTL results presented correspond to the 371 animals that passed all quality-filtering criteria, consisting of removal of genome-wide expression outliers based on principal component analysis [30], nominal genotype call rate (<0.95), and other quality metrics.

## Additional files

Additional file 1: Supplementary Materials. This document contains supplementary Tables S1-S5 and supplementary Figures S1-S5. (DOCX 669 kb)

Additional file 2: ChiSqTests. This spreadsheet contains chi-squared tests to determine if there is greater overlap than expected by chance between QTL for milk production traits and QTL for milk composition traits. (XLSX 16 kb)

## Abbreviations

eQTL: Expression quantitative trait locus
GEBV: Genomic estimated breeding value
RNASeq: RNA sequence data
