# Supplementary material for: Leveraging genetically simple traits to identify small-effect variants for complex phenotypes
Source: BMC Genomics. 2016 Nov 3;17:858. doi: 10.1186/s12864-016-3175-3 (PMC5094043; doi:10.1186/s12864-016-3175-3)
Supplement: Supplementary file 1 — Supplementary Materials. This document contains supplementary Tables S1-S5 and supplementary Figures S1-S5. (DOCX 669 kb) [file 12864_2016_3175_MOESM1_ESM.docx]

Supplementary Material

**Leveraging genetically simple traits to identify small-effect variants for complex phenotypes**

K.E. Kemper^1^, M.D. Littlejohn^2,3^, T. Lopdell^2,3^, B.J. Hayes^4,5,6^, L.E. Bennett^7^, R.P. Williams^7^, X.Q. Xu^7^, P.M Visscher^8^, M.J. Carrick^9^ & M.E. Goddard^1,4^

^1^Faculty of Veterinary and Agricultural Sciences, University of Melbourne, Royal Parade, Parkville Victoria 3052, Australia.

^2^Livestock Improvement Corporation, Cnr Ruakura and Morrinsville Roads, Newstead, Hamilton 3240, New Zealand.

^3^School of Biological Sciences, University of Auckland, 3A Symonds Street, Auckland 1010, New Zealand.

^4^Department of Economic Development, Jobs, Transport & Resources, AgriBio, 1 Park Drive, Bundoora Victoria 3086, Australia.

^5^Dairy Futures co-operative Research Centre, AgriBio, 1 Park Drive, Bundoora Victoria 3086, Australia.

^6^La Trobe University, AgriBio, 1 Park Drive, Bundoora Victoria 3086, Australia.

^7^CSIRO Agriculture and Food, Sneydes Road, Werribee Victoria 3030, Australia.

^8^Queensland Brain Institute, University of Queensland, St Lucia Queensland 4072, Australia.

^9^Berghan Carrick Consulting, Moonee Ponds 3039, Australia.

**Table S1.** Milk sugar, mineral and protein concentrations examined for QTL which overlap with milk production phenotypes.

|  | milk component phenotype | phenotype classification | abv. | unit | N_1_ | mean | s.d. | N_2_ |
| --- | --- | --- | --- | --- | --- | --- | --- | --- |
| 1 | lactose | sugar | lactose% | % | 1370 | 4.97 | 0.18 | 440 |
| 2 | calcium | mineral | Ca | mg/kg | 1373 | 1074 | 125.7 | 439 |
| 3 | potassium | mineral | K | mg/kg | 1373 | 1590 | 204.5 | 439 |
| 4 | magnesium | mineral | Mg | mg/kg | 1373 | 97.24 | 12.75 | 439 |
| 5 | sodium | mineral | Na | mg/kg | 1373 | 330.6 | 66.29 | 439 |
| 6 | phosphorus | mineral | P | mg/kg | 1373 | 891 | 92.66 | 439 |
| 7 | sulphate | mineral | S | mg/kg | 1373 | 300.3 | 33.22 | 439 |
| 8 | zinc | mineral | Zn | mg/kg | 1373 | 3.52 | 0.79 | 439 |
| 9 | lactoperoxidase | protein | lacP | mg/g | 1378 | 0.1 | 0.11 | 444 |
| 10 | lactoferrin | protein | lacF | mg/g | 1378 | 0.21 | 0.15 | 444 |
| 11 | ImmunogobulinG | protein | IgG | mg/g | 1378 | 0.39 | 0.18 | 444 |
| 12 | alpha-lactalbumin | protein | aLA | mg/g | 1378 | 0.82 | 0.2 | 444 |
| 13 | beta-lactoglobulin | protein | bLG | mg/g | 1378 | 2.87 | 1.23 | 444 |
| 14 | kappa-casein | protein | kCN | mg/g | 1378 | 3.46 | 1.01 | 444 |
| 15 | alpha-S1-casein | protein | aCN | mg/g | 1378 | 12.64 | 1.97 | 444 |
| 16 | beta-casein | protein | bCN | mg/g | 1378 | 11.77 | 2.07 | 444 |

N_1_ = total number of records per trait, N_2_ = final number of cows with genotypes and trait-deviations. Note that the all cows included in N_2_ had two records per individual and final phenotypes were the average of these two records (after corrections for non-genetic effects).

**Table S2.** Trait effects and significance level (p-value) of the three variants identified as highly associated with expression of SLC37A1 (solute carrier family 37 (glucose-6-phosphate transporter), member 1).

| SNP name | alleles  (ref > alt) | Position  (BTA, bp) | trait | trait  effect | P-value | σ^2^_P_ |
| --- | --- | --- | --- | --- | --- | --- |
| rs109400623^#^ | T > G | Chr1:144372020 | eSLC37A1 | 0.161 | 2.18x10^-18^ | 0.228 |
| rs208161466 | A > G | Chr1:144377960 | phosphorus conc. | 39.8 | 2.36x10^-11^ | 0.104 |
|  |  |  | eSLC37A1 | 0.161 | 2.18x10^-18^ | 0.228 |
|  |  |  | multi-trait - Holstein | - | 4.34x10^-11^ | - |
|  |  |  | milk yield – Holstein cows | -37.7 | 1.66x10^-3^ | 0.001 |
|  |  |  | milk yield – Holstein bulls | -37.2 | 5.14x10^-3^ | 0.003 |
|  |  |  | milk yield – Jersey cows | -42.4 | 5.70x10^-3^ | 0.002 |
|  |  |  | fat% - Holstein cows | 0.0224 | 3.21x10^-4^ | 0.001 |
|  |  |  | fat% - Holstein bulls | 0.0174 | 1.89x10^-2^ | 0.001 |
|  |  |  | fat% - Jersey cows | 0.0142 | 0.217 | 0 |
|  |  |  | protein% - Holstein cows | 0.0098 | 1.38x10^-4^ | 0.002 |
|  |  |  | protein% - Holstein bulls | 0.0103 | 2.28x10^-3^ | 0.003 |
|  |  |  | protein% - Jersey cows | 0.0108 | 0.0454 | 0.001 |
| rs109254133 | T > C | Chr1:144367474 | phosphorus conc. | 41.8 | 1.10x10^-11^ | 0.107 |
|  |  |  | eSLC37A1 | 0.160 | 3.55x10^-18^ | 0.224 |
|  |  |  | multi-trait - Holstein | - | 1.15x10^-11^ | - |
|  |  |  | milk yield – Holstein cows | -37.6 | 2.19x10^-3^ | 0.001 |
|  |  |  | milk yield – Holstein bulls | -40.3 | 3.17x10^-3^ | 0.003 |
|  |  |  | milk yield – Jersey cows | -45.2 | 3.26x10^-3^ | 0.002 |
|  |  |  | fat% - Holstein cows | 0.024 | 2.01x10^-4^ | 0.002 |
|  |  |  | fat% - Holstein bulls | 0.018 | 0.0212 | 0.001 |
|  |  |  | fat% - Jersey cows | 0.016 | 0.179 | 0 |
|  |  |  | protein% - Holstein cows | 0.010 | 7.54x10^-4^ | 0.002 |
|  |  |  | protein% - Holstein bulls | 0.011 | 1.61x10^-3^ | 0.003 |
|  |  |  | protein% - Jersey cows | 0.011 | 0.0376 | 0.001 |

^#^variant not identified in the 1000 bulls dataset (i.e. datasets 5 & 6, Table S5) and therefore not imputed into the animals measured for component milk traits or milk production.

**Table S3.** Conditional trait effects, when fitting rs209251505, and significance level (p-value) for the variant most highly associated with the multi-trait meta-analysis and highly associated α_s1_-casein concentration.

| SNP name | alleles  (ref > alt) | Position  (BTA, bp) | trait | trait  effect | P-value | σ^2^_P_ |
| --- | --- | --- | --- | --- | --- | --- |
| rs109193501 | A > G | Chr6:87154594 | α_s1_-casein conc. | -0.621 | 1.97x10^-5^ | 0.040 |
|  |  |  | multi-trait - Holstein | - | 1.00x10^-26^ | - |
|  |  |  | milk yield – Holstein cows | 3.98 | 0.809 | 0 |
|  |  |  | milk yield – Holstein bulls | -24.6 | 0.199 | 0 |
|  |  |  | milk yield – Jersey cows | 24.2 | 0.095 | 0 |
|  |  |  | protein yield - Holstein cows | -1.89 | 4.53x10^-5^ | 0.002 |
|  |  |  | protein yield - Holstein bulls | -2.73 | 1.09x10^-7^ | 0.009 |
|  |  |  | protein yield - Jersey cows | -1.44 | 0.001 | 0.002 |
|  |  |  | protein% - Holstein cows | -0.027 | 1.68x10^-14^ | 0.007 |
|  |  |  | protein% - Holstein bulls | -0.026 | 6.10x10^-8^ | 0.009 |
|  |  |  | protein% - Jersey cows | -0.044 | 1.08x10^-18^ | 0.020 |

**Table S4.** Conditional trait effects, when fitting rs109193501, and significance level (p-value) of the seven variants identified as highly associated with expression of CSN3 (kappa-casein).

| SNP name | alleles  (ref > alt) | Position  (BTA, bp) | trait | trait  effect | P-value | σ^2^_P_ |
| --- | --- | --- | --- | --- | --- | --- |
| rs459618023^#^ | A > G | Chr6:87386170 | eCSN3^*^ | 0.336 | 4.46X10^-22^ | 0.285 |
| rs382107247^#^ | CA > TG | Chr6:87387869 | eCSN3^*^ | 0.336 | 4.46X10^-22^ | 0.285 |
| rs134221650^#^ | T > C | Chr6:87392665 | eCSN3^*^ | 0.323 | 9.26X10^-22^ | 0.280 |
| rs136843602 | C > T | Chr6:87392674 | κ-casein conc. | -0.462 | 2.09x10^-9^ | 0.082 |
|  |  |  | eCSN3^*^ | -0.323 | 9.26X10^-22^ | 0.280 |
|  |  |  | multi-trait - Holstein | - | 6.89x10^-4^ | - |
|  |  |  | milk yield – Holstein cows | 15.4 | 0.323 | 0 |
|  |  |  | milk yield – Holstein bulls | 23.7 | 0.210 | 0 |
|  |  |  | milk yield – Jersey cows | 7.7 | 0.734 | 0 |
|  |  |  | protein yield - Holstein cows | -0.428 | 0.328 | 0 |
|  |  |  | protein yield - Holstein bulls | -0.177 | 0.725 | 0 |
|  |  |  | protein yield - Jersey cows | -0.414 | 0.557 | 0 |
|  |  |  | protein% - Holstein cows | -0.011 | 3.81x10^-4^ | 0.001 |
|  |  |  | protein% - Holstein bulls | -0.013 | 7.84x10^-3^ | 0.002 |
|  |  |  | protein% - Jersey cows | -0.012 | 0.104 | 0 |
| rs133766898 | C > T | Chr6:87400728 | κ-casein conc. | 0.462 | 2.20x10^-9^ | 0.082 |
|  |  |  | eCSN3^*^ | 0.324 | 2.16X10^-21^ | 0.274 |
|  |  |  | multi-trait – Holstein | - | 7.28x10^-4^ | - |
|  |  |  | milk yield – Holstein cows | -15.2 | 0.328 | 0 |
|  |  |  | milk yield – Holstein bulls | -23.4 | 0.216 | 0 |
|  |  |  | milk yield – Jersey cows | -5.6 | 0.788 | 0 |
|  |  |  | protein yield - Holstein cows | 0.433 | 0.323 | 0 |
|  |  |  | protein yield - Holstein bulls | 0.182 | 0.718 | 0 |
|  |  |  | protein yield - Jersey cows | 0.429 | 0.510 | 0 |
|  |  |  | protein% - Holstein cows | 0.011 | 3.87x10^-4^ | 0.001 |
|  |  |  | protein% - Holstein bulls | 0.012 | 8.14x10^-3^ | 0.002 |
|  |  |  | protein% - Jersey cows | 0.011 | 0.104 | 0 |
| rs209251505 | A > G | Chr6:87406609 | κ-casein conc. | 0.347 | 4.66x10^-9^ | 0.078 |
|  |  |  | eCSN3^*^ | 0.337 | 3.33X10^-22^ | 0.287 |
|  |  |  | multi-trait - Holstein | - | 1.18x10^-8^ | - |
|  |  |  | milk yield – Holstein cows | -21.2 | 0.106 | 0 |
|  |  |  | milk yield – Holstein bulls | -35.8 | 0.021 | 0.001 |
|  |  |  | milk yield – Jersey cows | -5.8 | 0.780 | 0 |
|  |  |  | protein yield - Holstein cows | 0.307 | 0.403 | 0 |
|  |  |  | protein yield - Holstein bulls | -0.052 | 0.900 | 0 |
|  |  |  | protein yield - Jersey cows | 0.423 | 0.516 | 0 |
|  |  |  | protein% - Holstein cows | 0.014 | 7.03x10^-7^ | 0.003 |
|  |  |  | protein% - Holstein bulls | 0.015 | 1.28x10^-4^ | 0.004 |
|  |  |  | protein% - Jersey cows | 0.011 | 0.103 | 0 |

^#^variants not identified in the 1000 bulls dataset (i.e. datasets 5 & 6, Table S5) and therefore not imputed into the animals measured for component milk traits or milk production.

^*^rs109193501 was not fitted as a covariate in the gene expression analysis.

**Table S5. Data summary and number of animals (N) available in each dataset.**

| Dataset origin | N | Breed of cattle  (sex, where applicable) | Data available |
| --- | --- | --- | --- |
| Australia | 444 | Holstein (cows) | 16 milk component traits + HD SNP^*,#^ |
| Australia | 8478 | Holstein (cows)^1^ | 5 milk production traits + HD SNP^*^ |
| Australia | 3049 | Holstein (bulls)^1^ | 5 milk production traits + HD SNP^*^ |
| Australia | 3917 | Jersey (cows)^1^ | 5 milk production traits + HD SNP^*^ |
| Worldwide | 280 | 1000 bull genomes^2^, Holstein | sequence variants in target regions |
| Worldwide | 61 | 1000 bull genomes^2^, Jersey | sequence variants in target regions |
| New Zealand | 371 | Holstein (cows)^3^ | mammary gland RNA sequence + HD SNP^*^ |
| New Zealand | 556 | pure & crossbred animals^4^  (including Holstein) | sequence variants in target regions |

^*^real and imputed high-density (HD) SNP genotypes from the Illumina BovineHD BeadChip.

^#^a total of 728 cows were measured for the component milk traits and used to make trait-deviations corrected for non-genetic effects for the final 444 genotyped cows which passed all quality controls.

Milk production traits are fat, milk and protein yield per lactation; and percentage of fat and protein in milk.

Component traits are milk lactose, mineral (calcium, potassium, magnesium, sodium, phosphorus, sulphur, zinc) and protein (lactoperoxidase, lactoferrin, immunoglobulinG, alpha-lactalbum, beta-lactoglobulin, kappa-casein, alpha-S1-casein, beta-casein) concentration.

**References**

1 Kemper, K. E. *et al.* Improved precision of QTL mapping using a nonlinear Bayesian method in a multi-breed population leads to greater accuracy for across-breed genomic predictions. *Genetics Selection Evolution* **47**, 29 (2015).

2 Daetwyler, H. D. *et al.* Whole-genome sequencing of 234 bulls facilitates mapping of monogenic and complex traits in cattle. *Nature Genetics* **46**, 858-865 (2014).

3 Littlejohn, M. D. *et al.* Expression variants of the lipogenic *AGPAT6* gene affect diverse milk composition phenotypes in *Bos taurus*. *PLoS ONE* **9**, e85757, (2014).

4 Littlejohn, M. D. *et al.* Functionally reciprocal mutations of the prolactin signalling pathway define hairy and slick cattle. *Nature Communications* **5**, (2014).


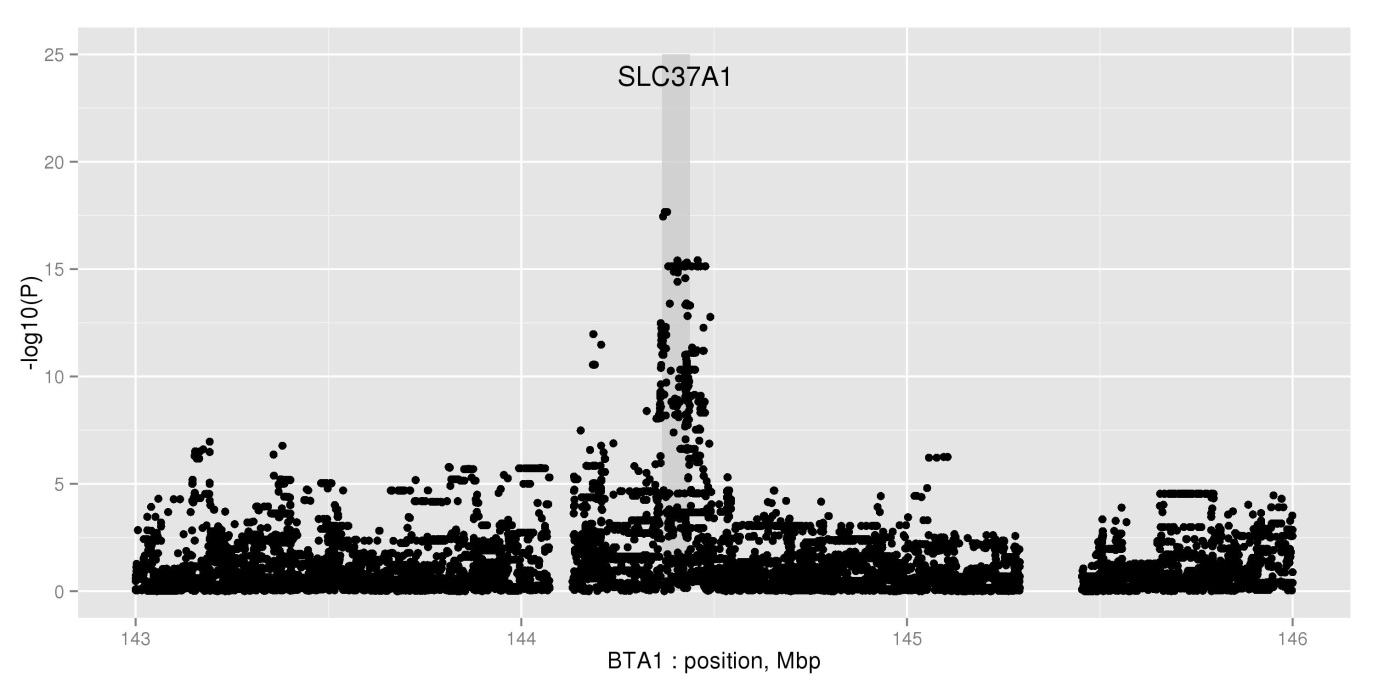


**Figure S1.** Association analysis of sequence variants with expression of SLC37A1.

| **a** | 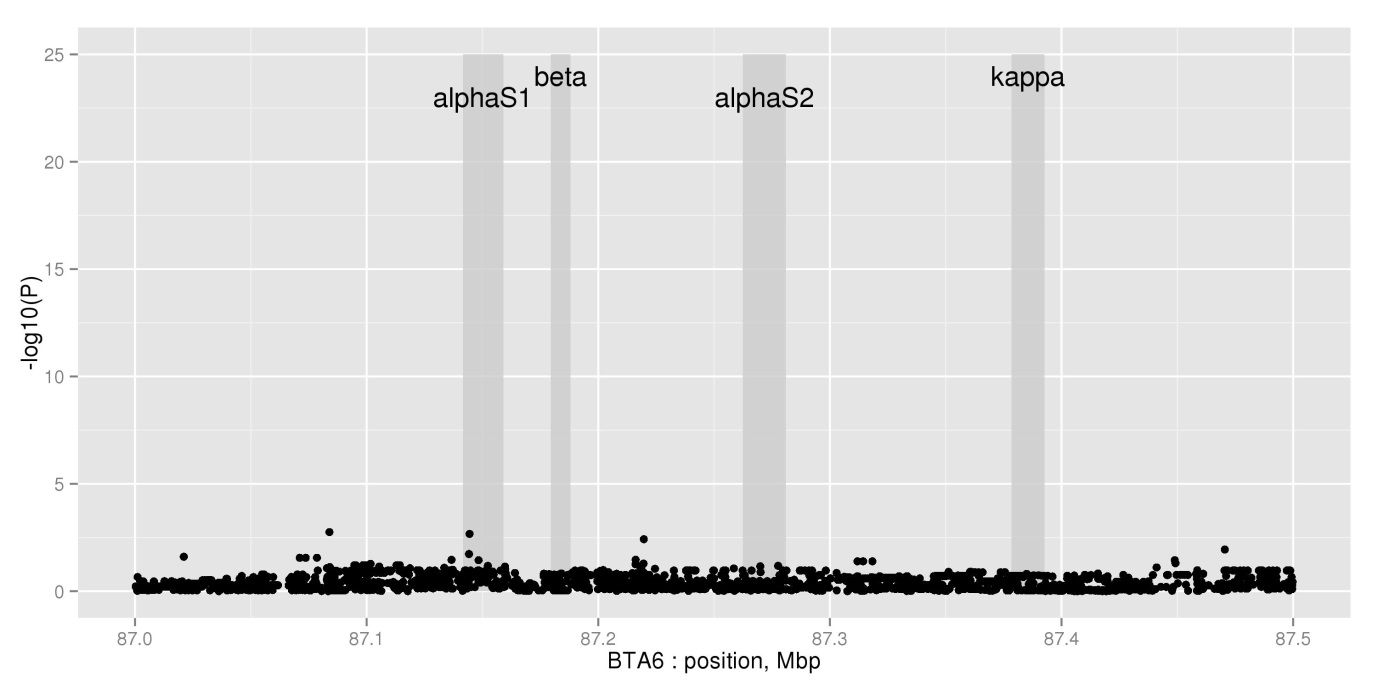 |
| --- | --- |
| **b** | 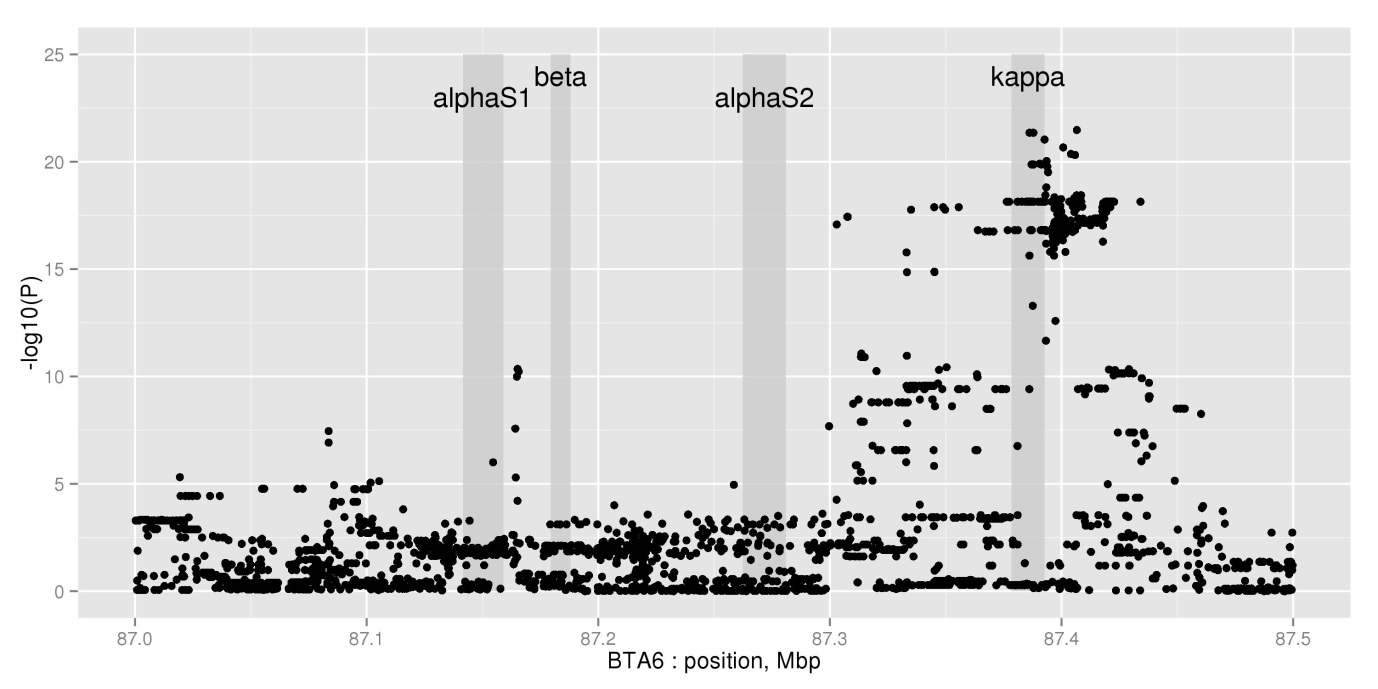 |

**Figure S2.** Association analysis of sequence variants with expression of (a) CSN1S1 (alphas1) and (b) CSN3 (kappa) casein.


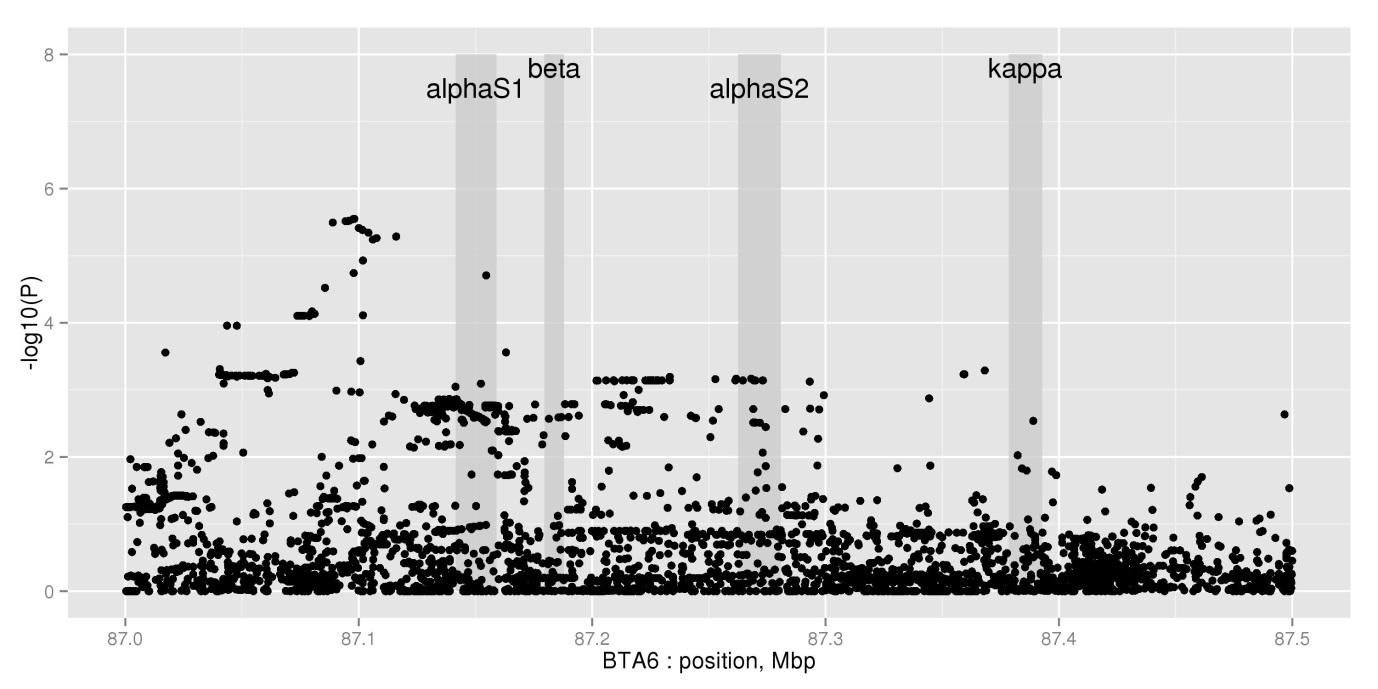


**Figure S3.** Association analysis of sequence variants with αS1-casein concentration fitting rs209251505 as a covariable.


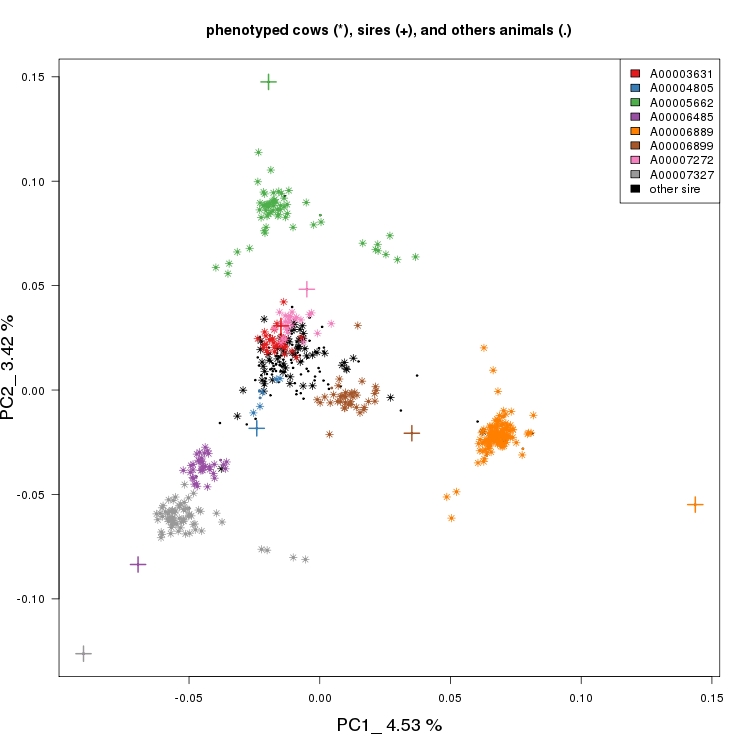
**Figure S4.** Principal component analysis for relationship matrix for 444 cows with measurements for 16 milk component traits and their genotyped ancestors. Cows with phenotypes are coloured by their sire (*) while sire genotypes are indicated by crosses (+).

**Figure S5.** Cumulative variance explained by the proportion of windows explaining the most amount of variance in milk composition traits. Traits are grouped into 3 broad groups of genetic architectures, Gp I: lactose%, lactoferrin, beta-lactoglobulin, calcium, phosphorus and kappa-casein; Gp II: potassium, zinc, lactoperoxidase, immunoglobulin-G, sodium and alpha-S1-casein; and Gp III: magnesium, sulphur, alpha-lactoalbum and beta casein.
